# Metastatic lung adenocarcinoma in a 20-year-old patient

**DOI:** 10.3747/co.v17i1.543

**Published:** 2010-02

**Authors:** O. Khan, W.P. Tong, N.J. Karlin

**Keywords:** Metastatic lung cancer, non-small-cell lung cancer, young patients

## Abstract

Lung cancer is rare disease in patients under 25 years of age. It typically occurs in older patients with a history of tobacco use. This case concerns a 20-year-old man with no history of tobacco use who complained of several months of cough and lower back pain and an 11.3-kg weight loss. He was treated for pneumonia after a chest radiograph showed total opacification of the right lung. Computed tomography imaging subsequently revealed a superior right hilar mass and mediastinal lymphadenopathy. Further imaging studies showed diffuse metastatic disease. Mediastinal biopsy showed poorly differentiated epithelioid tumour with desmoplastic stromal reaction, neutrophil infiltration, and squamous differentiation. Tissue immunostaining confirmed a non-small-cell lung cancer. Unfortunately, despite aggressive therapy, the patient’s disease progressed, and he died within 9 months. In this paper, we hope to illustrate the unique challenges in diagnosing and treating young patients with metastatic lung cancer.

## 1. INTRODUCTION

Lung cancer is the leading cause of death for men and women in the United States, surpassing deaths from breast, prostate, and colon cancer 1. The age-adjusted incidence for 2006 reveals that lung cancer is the number one cause of death for men over the age of 40 and for women over the age of 60^1^. Increasing age and tobacco use constitute the strongest risk factors for lung cancer. Young patients under 25 years of age without a history of tobacco use, environmental exposures, or genetic predisposition are only rarely diagnosed with lung cancer. Non-small-cell lung cancer (nsclc) typically presents at a more advanced stage and carries a poor prognosis.

## 2. CASE DESCRIPTION

**FIGURE 1:**
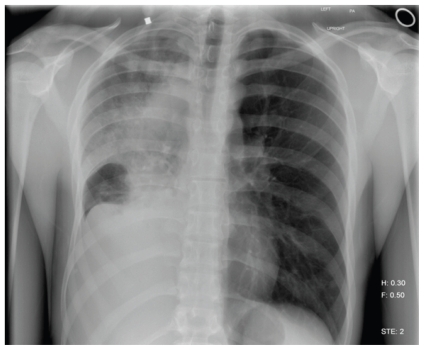
Posteroanterior view of the chest demonstrates complete opacification of the right hemithorax.

A 20-year-old man with no history of tobacco use presented with a several-months’ history of cough and lower back pain, and an 11.3-kg weight loss. Because of the persistent cough and development of hemoptysis, further imaging studies were obtained. A chest radiograph revealed total opacification of the right lung (Figure 1).

The patient was diagnosed with pneumonia and was started on antibiotics, but he did not improve.

**FIGURE 2:**
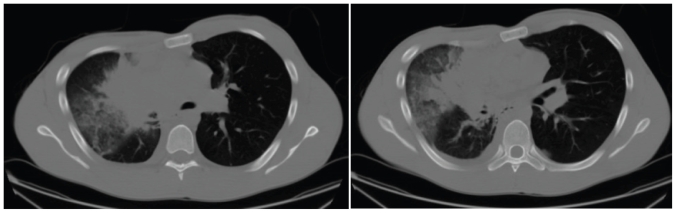
Computed tomography imaging of the thorax, with contrast, reveals a poorly defined 7×7×8-cm superior hilar mass.

Infectious serologies were negative. Computed tomography imaging of the thorax revealed a 7×7×8- cm mass in the superior right hilum, total collapse of the right lung with post-obstructive atelectasis, and mediastinal lymphadenopathy (Figure 2). Further imaging revealed retroperitoneal lymphadenopathy, renal and pancreatic masses, skeletal metastases in the pelvis and vertebral bodies, and intraparenchymal brain metastases. Interestingly, both adrenal glands were spared.

**FIGURE 3:**
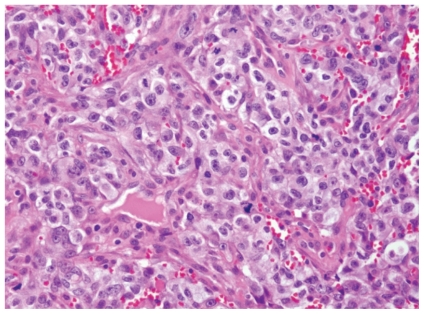
Biopsy of the mediastinal mass shows a poorly differentiated carcinoma, non-small-cell type. (Courtesy Kevin Leslie, md, of the Department of Pathology, Mayo Clinic Arizona; with permission)

Placement of a right bronchial stent and mediastinoscopy with biopsy were performed. The biopsy revealed poorly differentiated epithelioid tumour with desmoplastic stromal reaction, neutrophil infiltration, and squamous differentiation (Figure 3). The tumour cells stained positive for epithelial membrane antigen, pancytokeratin, thyroid transcription factor 1, and cytokeratins 8 and 7. Testing for epidermal growth factor receptor mutation and amplification was negative. Isochrome 12p was not detected. Serum β–human chorionic gonadotropin was 3.6 IU/L (normal range: 0–0.6 IU/L) and alpha fetoprotein was 1.5 ng/mL (normal reading: <6 ng/mL). Testicular ultrasound was unremarkable. These findings confirmed a primary lung adenocarcinoma.

The patient was urgently treated with cisplatin and etoposide. Radiotherapy was also initiated to lung, spine, and pelvis. He improved clinically, but required several hospitalizations throughout chemotherapy. Ultimately, his disease progressed, and he died within 9 months of the initial diagnosis.

## 3. DISCUSSION

Lung cancer is the leading cause of cancer-related death worldwide, with nsclc accounting for 85% of all lung cancers 2. Lung adenocarcinoma has the highest incidence among lung cancer patients, with a sex-specific incidence of about 30% in men and 37% in women in the United States 3. The strongest risk factors for lung cancer are tobacco use and age, although small-cell lung cancer and squamous cell lung cancer have a stronger association with tobacco use than does lung adenocarcinoma 3.

In patients under 25 years of age, nsclc is exceedingly rare, having an incidence rate for 2002–2006 of 0.3 per 100,000^4^. The most common types of lung cancer in this group of patients are pleuropulmonary blastoma, germ-cell tumours (teratocarcinoma), carcinoid, and metastatic cancer from a non-lung primary 5. In reference to nsclc occurring in young people, a higher incidence of adenocarcinoma is seen in female patients, and most cases show no history of tobacco use 6. This observation suggests that genetic factors may have a greater role in the development of cancer in this patient population. Genetic factors are known to play a role in the development of lung adenocarcinoma, and familial genetic clustering of lung cancer has been found. Common gene mutations in *KRAS, EGFR,* and *TP53* have been associated with a higher risk for development of lung adenocarcinoma. We do not know the *KRAS* status for this patient’s tumour.

Survival in this group of patients remains highly variable. Mizushima *et al.* found no difference in survival between lung adenocarcinoma patients less than and more than 30 years of age 6. Similarly, a retrospective study of patients younger than 50 years of age compared with those older than 50 found no difference in survival or in time to disease progression 9. In contrast, two other studies found a worse prognosis for young patients with lung adenocarcinoma than for their older counterparts 10.

Because of the dearth of cases, data evaluating the effectiveness of treatment for lung cancer in patients under 25 years of age are limited. Combined modalities of chemoradiation and surgical resection have been tried and compared. Bourke *et al.* studied lung cancer in patients less than 45 years of age, comparing them with patients more than 45 years of age at three different geographic sites 11. In that study, lung cancer staging was demonstrated to be the factor most determinant for survival in patients less than 45 years of age 11.

## 4. CONCLUSIONS

Metastatic nsclc is rare in patients under the age of 25 years. Additional malignancies to be considered for thoracic masses in this age group include germ cell tumours (teratocarcinoma), lymphoma, carcinoid, and metastases from a non-lung primary cancer. The prognosis of metastatic nsclc in young patients remains dismal. Further clinical trials evaluating this group of patients with metastatic nsclc are desperately needed.
